# Electrochemical analysis of uric acid excretion to the intestinal lumen: Effect of serum uric acid-lowering drugs and 5/6 nephrectomy on intestinal uric acid levels

**DOI:** 10.1371/journal.pone.0226918

**Published:** 2019-12-31

**Authors:** Kyoko Fujita, Hiroki Yamada, Masahiro Iijima, Kimiyoshi Ichida

**Affiliations:** Department of Pathophysiology, Tokyo University of Pharmacy and Life Sciences, Horinouchi, Hachioji, Tokyo, Japan; University Medical Center Utrecht, NETHERLANDS

## Abstract

Recently, extensive efforts have been made to understand the importance of the extra-renal uric acid (UA) excretion pathways and their contribution to UA-related diseases. However, the method typically used to measure UA concentrations in the intestinal lumen is difficult to real time and dynamic analysis. In this study, UA excretion in the rat intestinal lumen was measured in real time using an electrochemical method. A sensitive electrode to detect UA was constructed using a gold electrode modified with a mixed self-assembled monolayer. Excretion rate of UA in the intestine was calculated using time course data. A decrease in UA excretion rate was observed in the intestine after administration of serum UA-lowering drugs (benzbromarone, febuxostat, and topiroxostat). Inhibition of ATP-binding cassette transporter G2 (ABCG2) which has been reported as an important exporter of UA was suggested by administration of these drugs. On the other hand, an increase in excretion rate of UA was observed in the intestine of 5/6 nephrectomy rats. Upregulation of mRNA expression of the UA transporter organic anion transporter OAT3, which is related to the secretion at the basal membrane, suggested an enhancement of UA excretion by ABCG2, a high-capacity UA exporter. Observed urate excretion dynamics and mRNA expression of UA transporters in the intestine upon administration of serum UA-lowering drugs and 5/6 nephrectomy improve our understanding of the underlying mechanisms of intestinal UA excretion.

## Introduction

Uric acid (UA) is the end product of purine metabolism in humans. Serum UA levels are determined by the balance between UA production and UA excretion rates. High serum UA levels, which are considered to be higher than 7 mg/dL, are diagnosed as hyperuricemia. This condition induces gout and accelerates the progression of renal and cardiovascular diseases [1–3]. On the other hand, UA exerts antioxidant activity in our body and many reports have shown a correlation between UA levels and reduced risk or favorable progression of neurogenerative diseases [4, 5].

Renal transport of urate (the ion form of UA predominantly found at physiological pH) is known to be the main urate excretion route. A number of urate transporters have been identified in renal proximal tubule cells [6]. Among these, urate transporter 1 (URAT1/SLC22A12) on the apical membrane [7] and glucose transporter 9 (GLUT9/SLC2A9) at the basolateral membrane [8] play key roles in UA reabsorption. On the other hand, adenosine 5’-triphosphate (ATP)-binding cassette (ABC) subfamily G member 2 (ABCG2) on the apical membrane is important in UA excretion [9]. Defects on the ABCG2 gene were shown to be associated with hyperuricemia and gout [10, 11].

It is commonly accepted that two thirds of the urate is excreted from the kidney into the urine [12]. The remaining one third is excreted via extra-renal excretion, mainly through the intestine. Recently, the importance of the extra-renal UA excretion pathways has been gathering attention. Vaziri and colleagues showed an increase in intestinal UA excretion in a rat model of chronic kidney disease, in which renal UA excretion is greatly impaired [13]. The impact of the urate transporter ABCG2 function in the intestine on the levels of serum UA has also been reported. Yano and colleagues showed that 5/6 nephrectomized rats exhibited lower excretion of UA in the urine and overexpression of ABCG2 in the ileum, while serum UA did not significantly increase [14]. Another study also hypothesized that the degree of ABCG2 dysfunction in the intestine strongly affects the severity of hyperuricemia [11]. In fact, *Abcg2-*knockout mice showed a reduction in intestinal UA excretion and an increase in serum UA levels; however, the urinary UA excretion was significantly increased in these mice [15]. Thus, it was suggested that the impaired intestinal UA excretion pathway is compensated by an increase in renal UA excretion.

Serum UA-lowering drugs for hyperuricemia and gout treatment fall into two categories: uricosuric agents and UA production inhibitors. The effect of serum UA-lowering drugs on the urate transport activity of ABCG2 has been reported [16]. The transport of [8-^14^C]-UA in ABCG2-expressing membrane vesicles indicated that the general serum UA-lowering drugs topiroxostat, benzbromarone, and febuxostat drastically inhibited the UA transport activity of ABCG2.

Further investigation on the excretion of UA to the intestinal lumen is required in order to fully understand the contribution of this UA excretion pathway to UA-related diseases; however, the general method used to measure UA excretion in the intestine requires the resection of intestinal specimens from animals and measurement of [^14^C] urate transport ex vivo [13, 15]. Even in in vivo experiments using the intestine loop model, the measurement of UA concentrations in the intestine is a laborious and time-consuming process [17]. In this study, we constructed an electrode system to detect changes in UA concentration in the rat intestine. This system is simple and does not require the use of labeled molecules or special detection instruments. A dynamic analysis of UA concentration in the rat intestine at steady state and after administration of different serum UA-lowering drugs or 5/6 nephrectomy was performed.

## Materials and methods

### 2.1. Materials

6-Ferrocenyl-1-hexanethiol (HS-C_6_-Fc) and 6-Hydroxy-1-hexanethiol (HS-C_6_-OH) were purchased from Dojindo Laboratories (Kumamoto, Japan). Febuxostat and benzbromarone were purchased from TCI chemicals (Tokyo, Japan), and Wako Pure Chemical Industries (Osaka, Japan), respectively. Topiroxostat were kindly provided form Sanwa Kagaku Kenkyusho Co., LTD (Nagoya, Japan). All other chemicals were commercial products of reagent grade.

### 2.2. Electrodes

Gold bead electrodes were prepared by melting φ = 0.2 mm gold wire 99.999 from Unique Medical Co., Ltd (Tokyo, Japan) in flame. The procedure for the preparation of clean gold bead electrodes has been previously reported [18]. The crystal state of gold surface were checked by oxidation reduction reaction in sulfuric acid solution. Prepared gold bead electrodes were soaked in an ethanol solution containing 1 mM alkanethiols at room temperature for more than 1h to form self-assembled monolayers (SAM). Mixed SAMs were prepared by immersing gold bead electrodes into mixed ethanol solutions of thiol with fixed ratio (HS-C_6_-Fc:HS-C_6_-OH = 1:3, 1:1 and 3:1), but SAM surface compositions were not determined.

Integrated devise with 0.8 mm diameter which was fixed Pt wide and Ag/AgCl wire was constructed by Unique Medical Co., Ltd (Tokyo, Japan). Gold bead electrode was fixed into the integrated devise for a three electrode measurement. Prepared measurement device was fixed in intestinal closed loop.

### 2.3. Animals

Male SD rat (10 week of age) and male SD 5/6 nephrectomy (Nx) (Severe) rat (10 week of age) were purchased from Japan SLC, Inc. (Shizuoka, Japan). Surgical resection of the upper and lower thirds of the left kidney was performed at 8 week of age, then a right subcapsular nephrectomy was performed at 9 week of age. The animals were housed at room temperature (24 degree) with relative humidity (55 ± 5%). They were fed a standard chow (CE-2, Clea Japan, inc., Tokyo, Japan) with free access to tap water and kept on a 12-h:12-h light-dark cycle. All studies were carried out in accordance with the Institutional animal care committee at the Tokyo University of Pharmacy and Life Sciences.

### 2.4. UA lowering drug administration and preparation of closed loop

Topiroxostat, febuxostat and benzbromarone were suspended in methylcellulose, because the solubility of these drugs are very low. The prepared UA lowering drug solution was orally administrated to rat (50 mg/kg). A group orally administrated only methylcellulose was used as sham group. In this study, to avoid data variation based on the administration, excess amount of drugs compared dose prescribed in each interview form were administrated to rats. After two hours of oral administration, closed loop was constructed in intestine detailed below, then UA excretion was measured with electrochemical method.

Rats were anesthetized using pentobarbital (20 mg/Kg) with intraperitoneal administration and kept isoflurane with inhalation anesthesia. A midline abdominal incision was made. The intestinal segment was manipulated in order to minimize any intestinal blood supply disturbances. The 10 cm upper portion of the cecum was hemocliped with metal clip (Natsume Seisakusho Co., Ltd), then end of the cecum was cut. In order to remove all intestinal contents, the small intestine was flushed with a saline. To avoid the effect of other molecule than UA in intestine on the measurement, the appropriate amount of saline for flash was investigated by HPLC measurement (PU-980 Intelligent HPLC pump and UV-970M UV Ditector (Hitachi High-Tech Science corp.) with Supelcosil LC-18DB column (Sigma-Aldrich Co. LLC.). Potential molecules which affect a current change in electrochemical measurement, for example ascorbic acid, were confirmed that it did not exist. End of the cecum was hemocliped with a plastic disposable clip (Natsume Seisakusho Co., Ltd), and slight upstream was tie off with suture (Natsume Seisakusho Co., Ltd). A slight downstream from the inlet was cut and electrode devise was inserted into intestine and fixed with suture. The constructed closed loop at ileum with electrode device (about 3 cm length) was filled with 0.2 mM phosphate buffer (pH 7.8) containing 0.3 mM potassium oxonate.

### 2.5. Electrochemical measurement

Cyclic voltammetry (CV) and amperometry were measured by ALS electrochemical analyzer (Model 802D, BAS Inc., Tokyo). Calibration curve of the constructed measurement device was demonstrated each time with CV by changing UA concentration. Obtained amperometric data in intestine for 200–1400 sec were used for a regression expression. The correlation coefficient was used as change in current per second. Change in UA concentration per second was calculated by using calibration curve of each electrode.

### 2.6. Quantification of mRNA expression of transporters at intestine

Ileum sample used the closed loop at were obtained after electrochemical measurement. The sample was freezed with liquid nitrogen and fractured with SK-mill (Funakoshi CO. Ltd., Tokyo). Total RNA was extracted by using NucleoSpin RNA Plus (Takara Bio Inc., Shiga). After measuring the total RNA concentration using NanoPhotometer P-330 (Implen, Germany), cDNA was generated from 500 ng total RNA with PrimeScript RT reagent kit (Perfect Real Time) (Takara Bio Inc., Shiga). Quantitative RT-PCR was performed using Thunderbird SYBR qPCR Mix (Toyobo CO., Ltd., Osaka) and StepOne^™^ (Applied Biosystems, California, USA). Primers are shown in Supporting Information (S1 Table). Expression was estimated by the -ΔΔCt method and normalized to that of glyceraldehyde-3-phosphate dehydrogenase (Gapdh).

### 2.7. Statistical analysis

Since data were not normally distributed, the values presented here correspond to the median for each group. Unpaired Student’s t-test was used to compare the control group and groups treated with drugs. A value of *P* < 0.05 was considered to be significant.

## Results

### 3.1. Electrode for detection of UA

**Fig 1:**
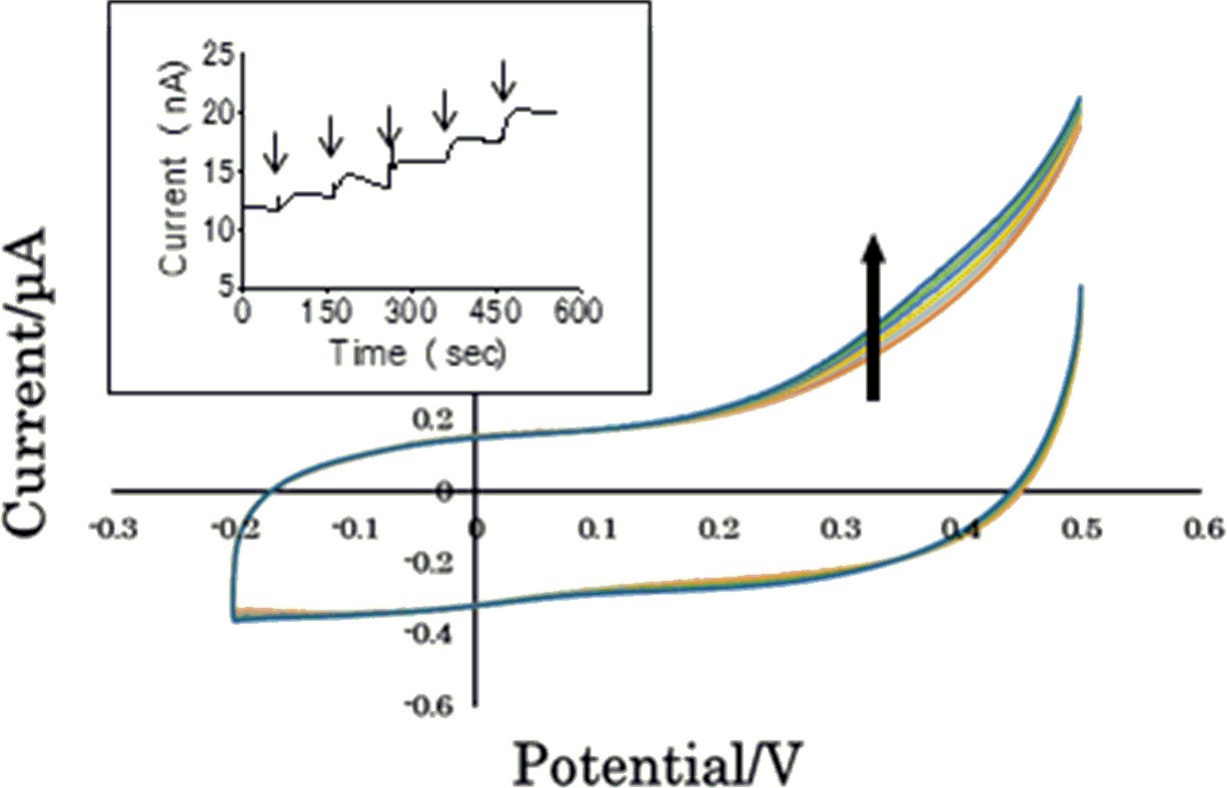
CV based on the constructed electrode modified with mixed SAM (HS-C_6_-Fc:HS-C_6_-OH = 1:3). Current change based on UA concentrations was measured in CV. UA concentration was changed by 0.1 μg/mL. Insert: Current change in amperometric measurement at 0.4 V. Addition of UA is indicated by arrows.

Gold bead electrodes modified with mixed SAM were set in the integrated device and current change based on UA concentration was measured in CV and amperometry. Fig 1 shows change in voltammograms around 0.4 V by addition of 0.1 μg/mL UA. The insert in this figure depicts the amperometric response by applying 0.4 V. Mixed SAM modified with HS-C_6_-Fc:HS-C_6_-OH = 1:3 showed the highest sensitivity of UA detection compared with SAM modified with HS-C_6_-Fc:HS-C_6_-OH = 1:1 and 3:1 (see S2 Table). The gold bead electrode (surface area of approximately 0.05 cm^2^) modified with mixed SAM (HS-C_6_-Fc:HS-C_6_-OH = 1:3) showed current change in 1.8 E-9 A by alteration of UA concentration in 0.1 μg/mL. Hereafter, the gold bead electrode modified with mixed SAM (HS-C_6_-Fc:HS-C_6_-OH = 1:3) was used for further investigation in this study.

### 3.2. Excretion rate of UA in the intestine

**Fig 2:**
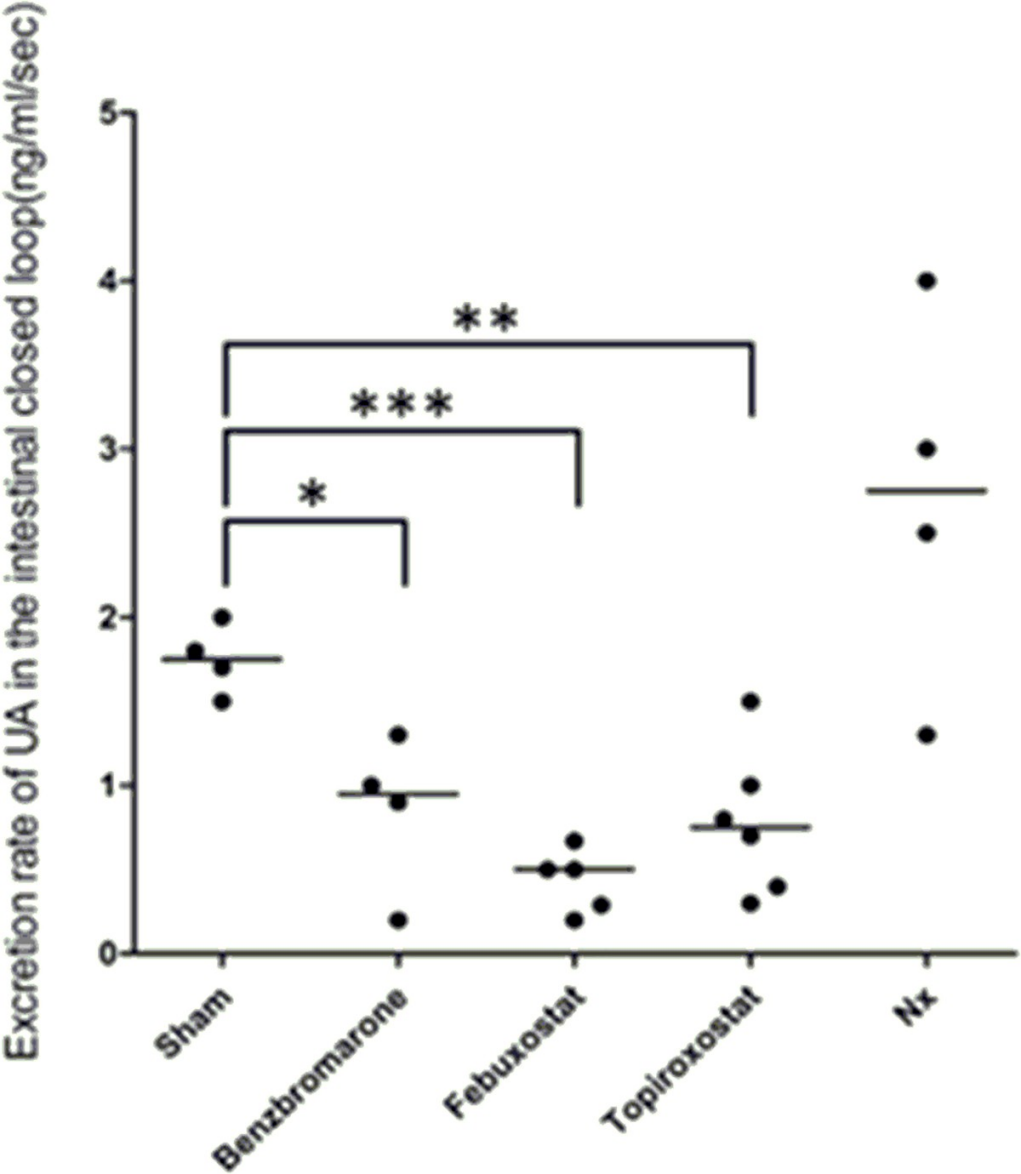
Excretion rate of UA in the intestinal closed loop. Calculated excretion rate of UA in the intestine of rats subjected to sham, administration of serum UA-lowering drugs (benzbromarone, febuxostat, and topiroxostat), or Nx (n = 4–6). Crossbar in each group shows the median. Significant differences between the sham group and other groups are indicated as * (p<0.05), ** (p<0.01), and *** (p<0.005).

By fixing the electrode device in the closed intestinal loop of naïve rats, a current increase with time was observed (S1 Fig in Supporting Information). The excretion rate of UA in the intestine was calculated based on the observed current change. Fig 2 shows the calculated excretion rate of UA in the intestine of rats in sham, serum UA-lowering drug (benzbromarone, febuxostat, and topiroxostat)-treated, and 5/6 nephrectomy (Nx) groups (n = 4–6). Administration of serum UA-lowering drugs significantly decreased the excretion rate of UA relatively to the sham group. On the other hand, the Nx group showed an increase in UA excretion rate compared with the sham group, but this difference was not significant. The median excretion rates of UA in the intestine of sham, benzbromarone-, febuxostat-, topiroxostat-treated, and Nx groups were 1.75, 0.95, 0.50, 0.75, and 2.75, respectively.

### 3.3. UA transporter mRNA expression in the intestine

**Fig 3:**
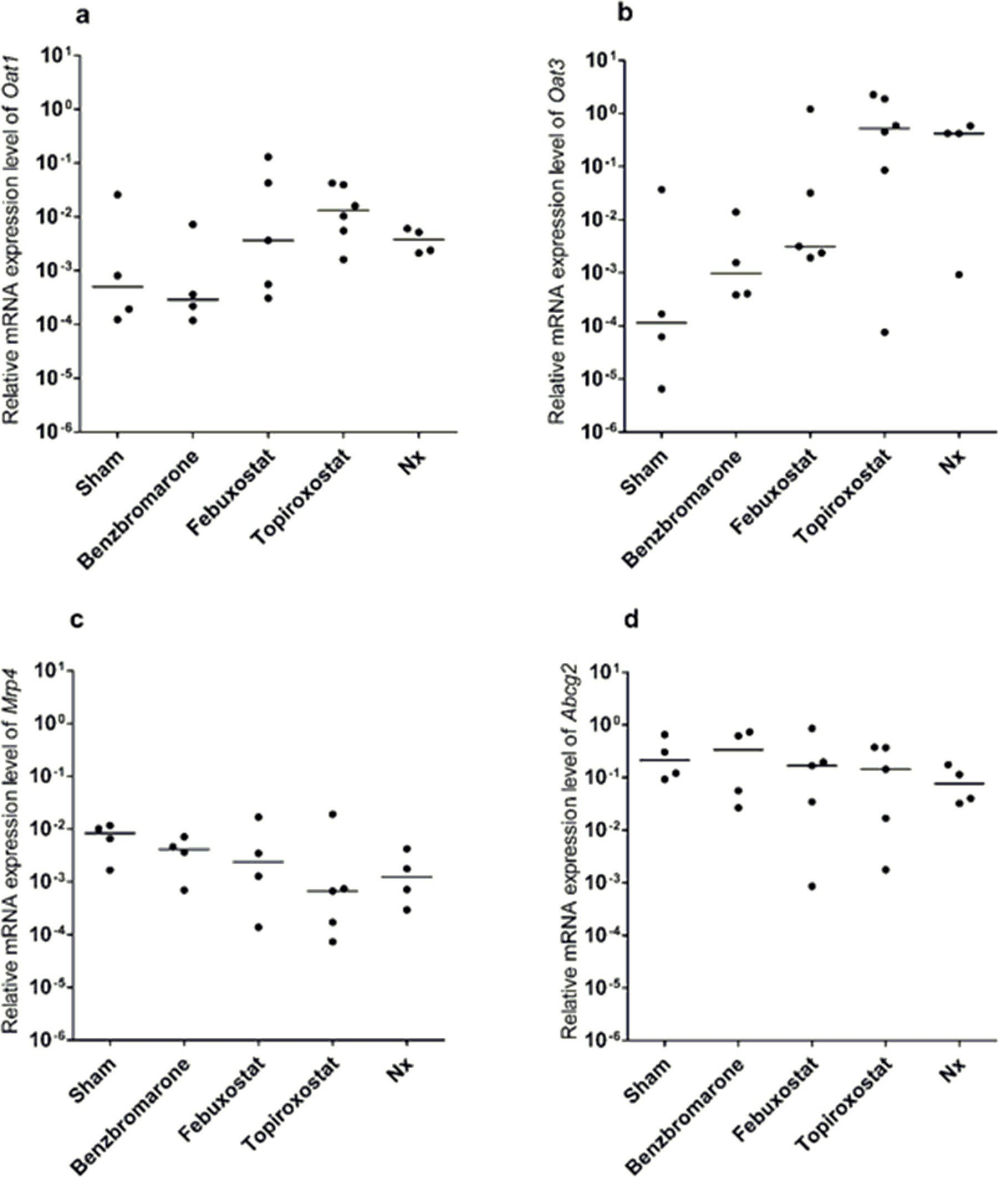
Relative mRNA expression of UA transporters in the intestine. mRNA expression of **a)** *Oat1*, **b)** *Oat3*, **c)** *Mrp4*, and **d)** *Abcg2* in the intestine of rats subjected to sham, Nx, or treated with serum UA-lowering drugs (benzbromarone, febuxostat, and topiroxostat) (n = 4–6). mRNA levels were normalized to *Gapdh*. Crossbar in each group shows the median.

Next, we investigated the mRNA levels of different transporters in the intestine. Although UA permeability in the intestine has been documented from the mucosa (absorption) and from the serosa to the mucosa, the majority of the transporters responsible for such permeability, particularly those on the basal membrane, are still unknown [19]. On the other hand, organic anion transporter 1 (Oat1) and organic anion transporter 3 (Oat3) were reported to be UA transporters located at the basal membrane of proximal tubule cells [20–22]. In the study conducted by Akazawa and colleagues, Oat1 and Oat3 were investigated as possible UA transporters in the intestine, though it was reported that the expression level of Oat1 and Oat3 in the intestine is not high compared to that of multidrug resistance protein 4 (Mrp4) and ABCG2 [23]. Among the seven transporters examined in this study that were reported to be UA transporters, four of them (*Oat1*, *Oat3*, *Abcg2*, and *Mrp4*) showed a clear amplification curve and were compared between groups (Fig 3). Relative mRNA expression level was normalized to *Gapdh*. Though no significant difference was found in the mRNA expression level of each transporter, some samples showed approximately 10^4^ times increase in the median value. In this study, we considered that there was a tendency for increase or decrease when the difference in the median of the mRNA expression level was over 10^2^ times. Relative mRNA expression of *Oat3* tended to increase in the intestine of rats treated with topiroxostat and subjected to Nx relatively to that observed in rats subjected to sham. On the other hand, the relative mRNA expression of *Abcg2* and *Mrp4* in the intestine of rats treated with serum UA-lowering drugs and those subjected to Nx showed no significant difference compared with that of the sham group. ABCG2 and MRP4 were reported to be UA transporters at the apical membrane in proximal tubules [24, 25].

## Discussion

### 4.1. Effect of UA-lowering drugs on UA excretion in the intestine

**Fig 4:**
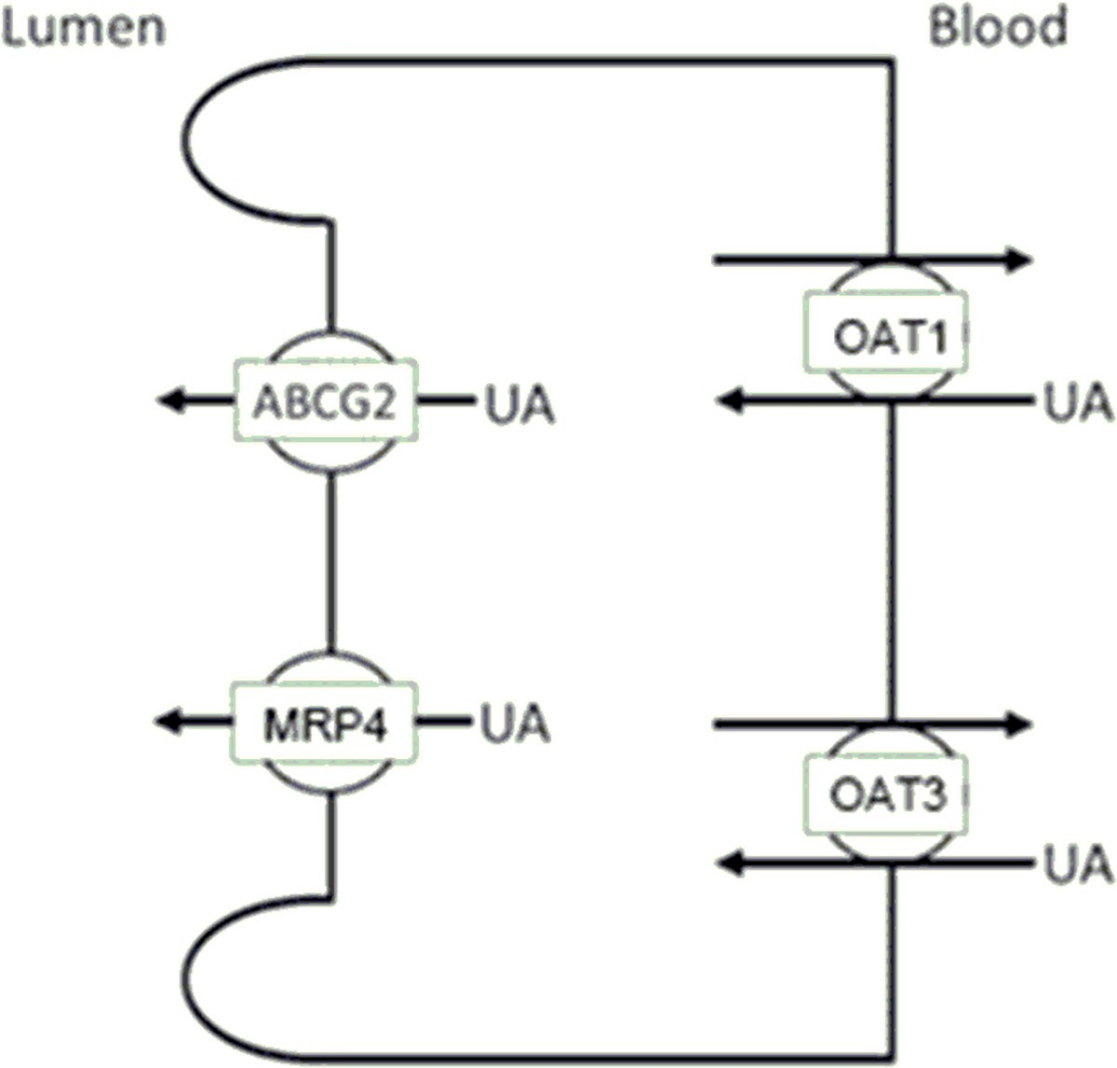
Proposed model for the action of UA-lowering drugs on intestinal UA transporters. Based on the results of this study, as well as previous reports, we suggest the expressed transporters Oat1, Oat3, Mrp4, and ABCG2 to be related to urate transport in the intestine. ABCG2 was inhibited by UA-lowering drugs used in this study.

The excretion rate of UA in the rat intestine after administration of serum UA-lowering drugs (benzbromarone, febuxostat, and topiroxostat) was decreased. Among these drugs, febuxostat was able to induce the most significant decrease (p<0.005). Febuxostat has shown to exert a strong inhibition of ABCG2, both in vivo and in vitro, and to induce an increase of the intestinal absorption of an ABCG2 substrate [16]. Benzbromarone is a general uricosuric agent that inhibits UA reabsorption in proximal tubules. Topiroxostat is an UA production inhibitor that blocks xanthine oxidoreductase (xanthine oxidase) the same way as febuxostat. These drugs have been reported to act as ABCG2 inhibitors [16]. ABCG2, known for its high activity as a UA exporter, has a calculated *K*_*m*_ of 8.2 mM related with the transport of UA [10]. Based on these evidences, we suggest that the observed decrease of UA excretion in the intestine is caused by inhibition of ABCG2 upon administration of benzbromarone, febuxostat, or topiroxostat (Fig 4). This is the first report that provides a real time analysis for the effects of serum UA-lowering drugs on intestinal excretion of UA in vivo.

Moreover, relative mRNA expression of *Oat3* in the intestine showed a tendency to increase in the groups treated with UA-lowering drugs, especially topiroxostat, compared with the sham group. Oat3 is known to be abundantly expressed on the basal membrane and excrete a wide variety of drugs and exogenous toxins, including uremic toxins [26]. Increase of *Oat3* mRNA expression after administration of UA-lowering drugs might suggest an increase in the excretion of these drugs. On the other hand, no significant changes were observed in relative mRNA expression of the examined transporters located in the apical membrane, MRP4 and ABCG2. These have been reported as efflux transporters for drugs and other molecules [25, 27]. Thus, it is suggested that the excretion capacity of these transporters was not saturated by the concentration of administrated UA-lowering drugs in the epithelial cells.

### 4.2. Effect of Nx on UA excretion in the intestine

In the present study, excretion rate of UA showed a tendency to increase in the Nx group compared with that of the sham group, though without statistical significance. It has been previously reported a significant decrease in urinary excretion of UA in Nx rats, but no significant differences were observed in serum UA levels in this case [14]. A compensatory increase of excretion in the intestine has been hypothesized, and increased expression of *Abcg2* in the ileum measured by real time PCR has been reported [14]. In our study, however, relative mRNA expression of *Abcg2* showed no significant changes. Probably, the portion of the intestine examined in our study presented different expression levels. Even in the literature, no significant difference in the expression of *Abcg2* was observed, except in the ileum, duodenum, jejunum, and transverse colon of Nx rats. Similarly to *Abcg2*, *Mrp4* showed no significant changes in mRNA expression in the intestine. MRP4 has been reported to be a transporter in UA excretion with a calculated *K*_*m*_ of 1.5 mM [24]. The lack of changes in relative mRNA expression of *Mrp4* and *Abcg2* suggests that the UA exporter capacity of MRP4 and ABCG2 was not saturated relatively to the UA concentration in epithelial cells. On the other hand, relative mRNA expression of *Oat3* showed a tendency to increase in the Nx group (approximately 10^4^ in median) compared with the sham group. OAT1 and OAT3 have been reported to be UA transporters contributing to its secretion at the basal membrane in proximal tubules [21]. Moreover, OAT3 has been shown to have a greater capacity to interact with anionic compounds than OAT1 [28]. The calculated *K*_*m*_ of OAT1 and OAT3 in the transport of UA were 0.9 mM and 2.9 mM, respectively [20, 29]. Increase of OAT3 in the intestine suggests an enhancement of UA excretion from the intestine. From the obtained results, the increase in UA excretion in the intestine of Nx rats is suggested to be dependent on OAT3, enhancing UA excretion by ABCG2, which is a high-capacity UA exporter.

In conclusion, a dynamic analysis of UA concentration in the rat intestine was achieved with an electrochemical method. Observed excretion rates of UA in the intestine were in agreement with the in vitro results reported in literature. Furthermore, the observed excretion dynamics of UA and the mRNA expression of UA transporters improve our understanding of the underlying mechanisms of intestinal excretion of UA. However, we consider that further examination is necessary to confirm the suggested mechanisms.

## Supporting information

S1 Fig Time course analysis of current change based on the UA excretion in the intestine of naïve rats. (DOCX)

S1 Table Forward and reverse primers for quantitative PCR (5′-3′). (DOCX)

S2 Table Current change per unit area of electrode by change in 0.1 μg/mL of UA. (DOCX)
