# Supplementary material for: Electrochemical analysis of uric acid excretion to the intestinal lumen: Effect of serum uric acid-lowering drugs and 5/6 nephrectomy on intestinal uric acid levels
Source: PLoS One. 2019 Dec 31;14(12):e0226918. doi: 10.1371/journal.pone.0226918 (PMC6938314; doi:10.1371/journal.pone.0226918)
Supplement: S1 Fig — (DOCX) [file pone.0226918.s001.docx]

**S1 Fig Timecourse of current change based on the UA excretion in intestine of naïve rat.**
