# Supplementary material for: Electrochemical analysis of uric acid excretion to the intestinal lumen: Effect of serum uric acid-lowering drugs and 5/6 nephrectomy on intestinal uric acid levels
Source: PLoS One. 2019 Dec 31;14(12):e0226918. doi: 10.1371/journal.pone.0226918 (PMC6938314; doi:10.1371/journal.pone.0226918)
Supplement: S1 Table — (DOCX) [file pone.0226918.s002.docx]

**S1 Table. Primers for quantitative PCR**

| Gene name | Forward (5’ to 3’) | Reverse (5’ to 3’) |
| --- | --- | --- |
| *Oat1/Slc22a6* | TCAGCAAAGATGGAGGTCTGG | TAAAGCGGAGGCAAGATTCG |
| *Oat3/Slc22a8* | CTGAAGGAGATGGCCCAGTC | CCAGGTCAGGATAGGCTTGC |
| *Mrp4/Abcc4* | CAGGGCTGCTGAATGCAATA | TTGGATTCGGGAAGACTGAGA |
| *Urat1/Slc22a12* | TGAGGATGGCTGGGTTTACG | CCAGCCAGGAAGATGGACTG |
| *Glut9/Slc2a9* | GCTTGCCCTAGCTTCCCTGA | AGGAAGGAGGACCCGAAGG |
| *Bcrp/Abcg2* | CCGGAAAACAGCTGAGAAAG | GAAATTGGCAGGTTGAGGTG |
| *NTP homologue/Slc17A4* | CTCCACTGACTCCCAGGGCT | CTGGGGATTGGAGCCAAGAATGAG |
| *Gapdh* | GGTGGACCTCATGGCCTACA | ATTGTGAGGGAGATCCTCAGTGT |
