# Supplementary material for: Electrochemical analysis of uric acid excretion to the intestinal lumen: Effect of serum uric acid-lowering drugs and 5/6 nephrectomy on intestinal uric acid levels
Source: PLoS One. 2019 Dec 31;14(12):e0226918. doi: 10.1371/journal.pone.0226918 (PMC6938314; doi:10.1371/journal.pone.0226918)
Supplement: S2 Table — (DOCX) [file pone.0226918.s003.docx]

**S2 Table. Current change per unit area of electrode by change in 0.1μg/ml of UA**

| HS-C_6_-Fc: HS-C_6_-OH* | Current change (A/cm^2^) |
| --- | --- |
| 1：1 | 3.6E-8 |
| 1：3 | 2.9E-7 |

*Current change was not observed when HS-C_6_-Fc: HS-C_6_-OH = 3:1
